# Major Problems in Clinical Psychological Science and How to Address them. Introducing a Multimodal Dynamical Network Approach

**DOI:** 10.1007/s10608-024-10487-9

**Published:** 2024-06-24

**Authors:** Marlon Westhoff, Max Berg, Andreas Reif, Winfried Rief, Stefan G. Hofmann

**Affiliations:** 1 Department of Psychology, Translational Clinical Psychology Group, Philipps-University of Marburg, Schulstraße 12, D-35032 Marburg, Germany; 2 Department of Psychology, Clinical Psychology and Psychotherapy Group, Philipps-University of Marburg, Gutenbergstraße 18, D-35032 Marburg, Germany; 3 Department of Psychiatry, Psychosomatic Medicine and Psychotherapy, Goethe University Frankfurt, University Hospital, D-60629 Frankfurt, Germany; 4 Fraunhofer Institute for Translational Medicine and Pharmacology ITMP, Frankfurt, Germany

**Keywords:** Network analysis, Mental disorders, Longitudinal data, Psychological treatment, Causal inference, Ecological momentary assessment

## Abstract

**Background:**

Despite impressive dissemination programs of best-practice therapies, clinical psychology faces obstacles in developing more efficacious treatments for mental disorders. In contrast to other medical disciplines, psychotherapy has made only slow progress in improving treatment outcomes. Improvements in the classification of mental disorders could enhance the tailoring of treatments to improve effectiveness. We introduce a multimodal dynamical network approach, to address some of the challenges faced by clinical research. These challenges include the absence of a comprehensive meta-theory, comorbidity, substantial diagnostic heterogeneity, violations of ergodicity assumptions, and a limited understanding of causal processes.

**Methods:**

Through the application of multimodal dynamical network analysis, we describe how to advance clinical research by addressing central problems in the field. By utilizing dynamic network analysis techniques (e.g., Group Iterative Multiple Model Estimation, multivariate Granger causality), multimodal measurements (i.e., psychological, psychopathological, and neurobiological data), intensive longitudinal data collection (e.g., Ecological Momentary Assessment), and causal inference methods (e.g., GIMME), our approach could improve the comprehension and treatment of mental disorders. Under the umbrella of the systems approach and utilizing e.g., graph theory and control theory, we aim to integrate data from longitudinal, multimodal measurements.

**Results:**

The multimodal dynamical network approach enables a comprehensive understanding of mental disorders as dynamic networks of interconnected symptoms. It dismantles artificial diagnostic boundaries, facilitating a transdiagnostic view of psychopathology. The integration of longitudinal data and causal inference techniques enhances our ability to identify influential nodes, prioritize interventions, and predict the impact of therapeutic strategies.

**Conclusion:**

The proposed approach could improve psychological treatment by providing individualized models of psychopathology and by suggesting individual treatment angles.

## Introduction

### Challenges for Contemporary Clinical Psychology

Despite steady development of novel psychological treatments, the field of clinical psychology faces substantial challenges. While clearly effective at a group-level, effect sizes for anxiety and depression treatments remain moderate (Cuijpers et al., 2019; Weitz et al., 2018), with substantial therapy dropout (Cooper & Conklin, 2015; Gersh et al., 2017), high non-responder rates (Springer et al., 2018) and some patient subgroups deteriorating despite treatment (Cuijpers et al., 2018). Furthermore, when compared to other fields of medicine (e.g., to oncology) psychotherapy has not made significant progress in developing more and more effective treatments in the last decades (S. C. Hayes & Hofmann, 2021). While there is high-quality evidence, e.g., from Great Britain that fortunately attests an increase in *practical effectiveness* over time for large therapy programs (Clark et al., 2018; Gyani et al., 2013; Saunders et al., 2020), this does not correspond to an increase in the *efficacy* of cognitive behavioral therapy itself. Further, for anxiety disorders, there seems to be meta-analytic evidence from randomized placebo-controlled trials (Bhattacharya et al., 2022; Carpenter et al., 2018; Hofmann & Smits, 2008) that effect sizes of psychological treatments may have been overestimated by earlier research. Judging from routine-care data and meta studies alike, there still is substantial room for improvement regarding psychological treatments.

In the modern debate, shortcomings in categorical diagnostic systems have repeatedly been discussed as a central reason for current treatment outcomes. This critique was introduced by proponents of more biomedical approaches (e.g., Insel, 2014; Insel et al., 2010) and later also by scientists who introduced new hierarchical “latent variable” models (e.g., Ringwald et al., 2023; Ruggero et al., 2019). And the argument was also forwarded by proponents of network models, with which they hope to obtain a better conceptualization of mental disorders to ultimately be able to better allocate patients based on the improved diagnostic criteria and to acquire a better description of the dynamic change processes during therapy (e.g., A. M. Hayes & Andrews, 2020). In this opinion paper, we will argue that a multimodal dynamical network approach (MMDNs) could help with improving clinical research and practice. Prior to this, we will provide a brief overview of problems in contemporary clinical research that we hope to address with the MMDNs approach.

### Five Problems for Clinical Psychological Science

#### Problem one: No overarching meta-theory.

Psychological science in general and therefore also clinical psychology lacks an overarching meta-theory for explaining *why* certain results emerge (e.g., Zagaria et al., 2020). Consequently, distinct and more importantly incompatible explanations are given for clinical phenomena. A meta-theory in the context of mental disorders can be defined as a theory that provides a unified framework for explaining how a large variety of mental disorders develop and maintain themselves. It should provide answers to big picture questions like: What is a mental disorder? Why do disorders occur and why do they persist? How and in which context did they develop? There seems to be currently too little emphasis regarding those “*why questions”* as well as regarding functional and developmental aspects of mental disorders. Or as Hayes et al. (2020a, p. 2) put it: “what are its functions, what are the mechanisms or processes involved in accomplishing these functions, how does the particular feature or trait develop, and what is its history?”

Currently, psychological theories often focus on a limited scope of explainable phenomena within a narrow range of problems. Although there are many psychopathology models for specific mental disorders, such as social anxiety, depression, psychosis, or post-traumatic stress disorder, there is a lack of comprehensive descriptions and conceptualizations of mental disorders in general. Because clinical psychology currently has no agreed-upon meta-theory of mental health and disorders, effective communication between experts of different clinical areas can become difficult (e.g., Goertzen, 2008) and direct model comparison is oftentimes hard (Colombo & Hartmann, 2017). As we will note further below, the Extended Evolutionary Meta Model (EEMM; Hayes et al., 2020), offers such a meta-theory by emphasizing the interconnected nature of individual symptoms, mental processes, and (neurobiological) mechanisms.

#### Problem two: Artificial comorbidity.

A problem inherent in current diagnostic systems like the Diagnostic and Statistical Manual 5th edition (DSM-5) (American Psychiatric Association, 2013) or the International Classification of Diseases 11th edition (ICD-11) (WHO, 2019) is the predominant emphasis on describing mental disorders as categorized, distinct entities (with noteworthy exceptions in the personality disorder section of the ICD-11: Krueger, 2013). However, clinical reality seldom adheres to these categories. When analyzed with dimension-reducing techniques (e.g., Kotov et al., 2017) or from a complex systems perspective (e.g., Heeren, Bernstein et al., 2018a; Heeren & McNally, 2018; McNally et al., 2017; Wasil et al., 2020) there seems to be considerable “comorbidity” between “different” disorders. Typically, categorical systems define mental disorders by specific sets of observable symptoms. This approach emphasizes (and maximizes) the outward (or phenotypic) differences between conditions, resulting in many distinct diagnoses. However, because many conditions often occur in tandem, concerns were raised that the categorical approaches may have led to an overemphasis on “distinct categories” that are variations of broader underlying syndromes (e.g., Andrews, 1996; Blashfield et al., 2014) (hence the term “artificial comorbidity”). Additionally, recent empirical and conceptual research suggests that many disorders share biological, psychological, and social process variables (e.g., Berg et al., 2022; Fledderus et al., 2010; Hakulinen et al., 2016; Krupnik, 2021; McEvoy & Mahoney, 2012) raising even more questions about the validity of current diagnostic systems. One reason for the modest efficacy of state-of-the-art psychological interventions may come from the fact that treatment development has largely corresponded to target discrete categorical diagnoses, failing to account for broader underlying mechanisms (e.g., rumination, anhedonia, emotion regulation problems, maladaptive expectations etc.). This was also shown empirically: Single-diagnosis protocols were suggested to produce diminished effects when multiple, “comorbid” disorders were present (e.g., Craske et al., 2007; Gibbons & DeRubeis, 2008; Steketee et al., 2001). Taken together, there is a plethora of evidence for a substantial overlap between diagnostic categories that are typically not represented in (most) treatment protocols, thereby probably limiting their impact.

#### Problem three: Heterogeneity within diagnostic categories.

In addition to the problem of artificial comorbidity, there is evidence that there is also substantial heterogeneity within diagnostic categories. For depression e.g., research using different methods indicates that this category does not represent a single, unified syndrome (Antonijevic, 2008; Fried & Nesse, 2015; Sullivan et al., 2002). Consequently, different patients with depression will present symptoms that are quite distinct for each individual patient. Treatment protocols are oftentimes designed to account for “typical” symptoms in a given diagnostic category and therefore have continued to fail to adapt to this considerable within-patient heterogeneity, diminishing the progress in developing effective psychotherapy protocols (Hayes et al., 2020).

#### Problem four: The ergodicity problem.

In order to be allowed to replace (mathematically complex) dynamical descriptions of a given system with a (mathematically simpler) probabilistic description of the same system, the ergodicity assumption is needed. It states that probabilistic simplifications of a given system are acceptable if it is ergodic:

- The process at hand is stationary: This means (I) that the mean of a process has to be constant in time; (II) the variance of the process has to be constant in time; and (III) that the dependencies characterizing the process *only* depend upon the relative distance between time points.
- The process at hand is homogeneous across subjects. This means that each subject in the population has to obey the same dynamic model (Molenaar, 2013; Peters, 2019).

Both assumptions are violated in psychological testing (Gomes et al., 2019) and in treatment research most of the time (Hayes et al., 2020). But clinical psychology nevertheless typically relies on data that was gathered from group samples with relatively few measurement timepoints. Frequently, we then apply this data to predict e.g., individual-level therapy success without considering violations of ergodicity. While in some cases, this procedure can be valid (for a brief discussion, see: Adolf & Fried, 2019) non-ergodic processes can pose a problem for therapy research. To summarize: Clinical psychology largely relies on probabilistic group-level descriptions of disorders but dynamical descriptions of psychopathology in individuals are needed to inform the detection of *individual processes* that cause and maintain psychopathology.

#### Problem five: No insight into causal processes.

Diagnostic systems like DSM-5 or ICD-11 present a list of criteria (symptoms) for defining each diagnostic category. While these lists offer utility for simplified diagnosis, they are *descriptive only* in the sense that they do not allow for the identification of mechanisms necessary for the maintenance / progression of a given disorder. In a network analysis of depression symptoms, Fried et al. (2016, p. 1) found that the symptoms of depression described in the DSM-5 are not necessarily the symptoms with a high degree of centrality, indicating that the DSM-5 symptoms are not always the ones that are expected to “influence many others and have […] causal influence” over the system. This highlights a common problem observed in other studies as well (for other examples see e.g.: Armour et al., 2017; Lazarov et al., 2020; Price et al., 2019). Current diagnostic systems cannot distinguish between symptoms that are mere “epiphenomena” of a given disorder and symptoms that have a causal relationship with disorder progression and maintenance.

### Advancing Mental Health Research through Multimodal Dynamical Network Analyses

To help overcome the barriers of traditional research in clinical psychology, we present an approach for using network analysis to foster psychological science. Our research agenda combines intensive *longitudinal data* collection (e.g., Curtiss et al., 2022; Hahn et al., 2021; Odenthal et al., 2023) from *multimodal sources* with state-of-the-art *network analysis* and *causal inference* methods (e.g., Shi et al., 2021; Zečević et al., 2021). MMDNs aim to integrate psychological processes (e.g., attention, expectations; e.g., Heeren, Jones et al., 2018b; Isvoranu et al., 2017; McNally et al., 2022), psychopathological symptoms (e.g., fear, listlessness, sleep disturbance; e.g., Barthel et al., 2020; Curtiss et al., 2018; Uhlhaas & Singer, 2015), and – whenever promising – neurobiological correlates (e.g., resting-state markers, neuromodulation, neurotransmitter systems; e.g., Cole et al., 2020; Kaiser et al., 2022; Kringelbach & Deco, 2020), providing a comprehensive, multimodal perspective on mental disorders (e.g., Taschereau-Dumouchel et al., 2022).

**Fig. 1:**
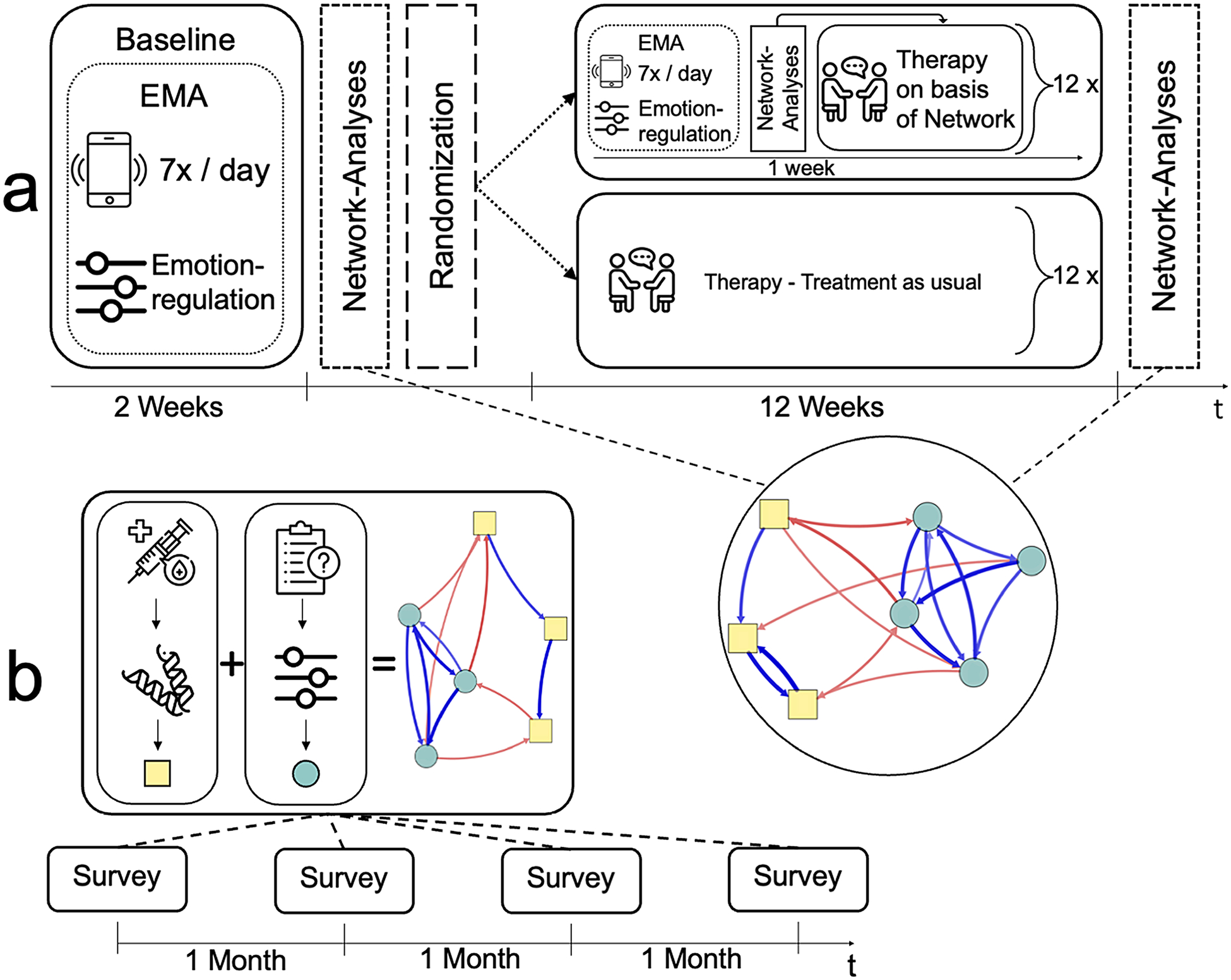
Two exemplary study designs based on multimodal dynamic network principles. An illustration of our approach with two worked-out examples. Depicted networks do not reflect real data, but are solely intended for illustrative purposes. Study A employs intensive data collection using EMA surveys, focusing on patients with emotion regulation difficulties. EMA survey is utilized to combine measures of both “normal” psychology and psychopathology. Baseline surveys establish individual dynamic networks based on nodes derived from the Difficulties In Emotion Regulation Scale (DERS; Gratz & Roemer, 2004), in this case, Emotional Awareness, Emotional Clarity, Acceptance, and Impulse Control (highlighted in circles). Transdiagnostic processes such as stress, physical activity, and rumination are included (highlighted in squares). Participants complete surveys seven times a day for two weeks. Randomly assigned to a “network-based therapy” or treatment-as-usual condition, participants in the network-based therapy condition receive tailored treatment based on their network model. Interventions particularly target critical self-sustaining feedback loops and pertinent (i.e., central) nodes. Throughout the 12-week therapy period, they continue responding to relevant questions using EMA. In the treatment-as-usual condition, therapy is manual-based without continuous data collection. At the end of therapy, networks are reconstructed for all participants for evaluation. Study B utilizes a panel model with less frequent data collection. Participants attend data collection sessions four times at monthly intervals. Blood samples are collected during each session using Enzyme-Linked Immuno-Sorbant Assay (ELISA) to detect inflammatory markers (C-reactive protein, interleukin, and fibrinogen) that were shown to be relevant to mental health and emotion regulation (e.g., Dedoncker et al., 2021; Fried et al., 2020). Immunoassays can be cost-prohibitive in some cases. However, considering the modest measurement density, a larger sample size could be aimed for. Participants also respond to emotion regulation questions using the DERS. Psychological and biological variables serve as nodes for network analyses. The presented study utilizes a Panel VAR design to analyze longitudinal associations between variables, differentiating within-subject and between-subject effects. Specifically, it employs cross-lagged panel models to investigate the multimodal relationship between inflammatory markers and symptoms of emotion regulation problems. The analysis explores temporal dynamics, reciprocal influences, and potential causal relationships. Valuable insights are gained regarding the impact of emotion regulation changes on biological markers. Additionally, the study aims to explore influential effects of variables such as gender, age, and disorder status and the relationship between inflammatory markers and symptoms of emotion regulation problems. Icons used in the figure come from the website Flaticon and are subject to the terms of the Creative Commons license (CC BY 4.0). Attribution and authorship credentials are detailed in the acknowledgment section and references, respectively

Longitudinal measurements are essential, for capturing the evolution of network states over time. By employing MMDNs (e.g., Morales et al., 2018; Stein et al., 2023; Wang et al., 2014; Yu et al., 2021), such as time-series or panel data analysis, we enable monitoring, prediction, and intervention planning for mental disorders. Data collection methods like Ecological Momentary Assessment (EMA; Myin-Germeys & Kuppens, 2021; Shiffman et al., 2008) and passive data collection techniques (e.g., smartphones, wearable sensors, electronic recordings, social media, and Internet data; Harari et al., 2016; Mohr et al., 2017) allow for data acquisition in natural environments, providing valuable insights into symptomatology development and dynamical interactions. These data can be supplemented with physiological data (e.g., Kamarck et al., 2003), neuroimaging (e.g., Seidel et al., 2022), or electroencephalography (e.g., Yap et al., 2022), enhancing the diagnostic and treatment capabilities of network analysis (for an exemplary illustration, see Fig. 1).

To analyze longitudinal networks, our approach aims to utilize various statistical methods, including autoregressive models (e.g., Bringmann et al., 2013; Epskamp, 2020), Group Iterative Multiple Model Estimation (GIMME; Gates & Molenaar, 2012), multivariate Granger causality (Granger, 1969), and Bayesian networks (e.g., McNally et al., 2017; Pearl & Mackenzie, 2019), and possibly advanced machine learning techniques such as deep neural networks (e.g., Goodfellow et al., 2016; Okuno & Woodward, 2021). These methods facilitate the identification of stable network connections (i.e., strength of connections) and contribute to improving treatment outcomes (McElroy et al., 2019).

Next, we discuss the potential of MMDNs in addressing and potentially overcoming the five central problems previously described. We will argue that our approach can help to enhance our understanding of mental health by modeling temporal processes and integrating within-person networks, accounting for heterogeneity, detecting group and individual effects, and identifying causal factors within disorders.

### Addressing Problem One. The Systems Approach as a Meta-Theory

Given the numerous and sometimes incompatible explanations for clinical phenomena that can hamper development in clinical science (Zagaria et al., 2020), the time may be ripe for a unifying meta-theory that provides a consistent framework for explaining the development and maintenance of a wide variety of mental disorders. The systems approach could provide such a unified framework. Building on previous work (e.g., Borsboom, 2017; Hofmann et al., 2016; McNally, 2021; Woods et al., 2020), we argue that mental disorders can be understood as intricate networks of mutually interacting symptoms. These symptoms do not merely reflect underlying diagnoses but describe general problems that encompass psychopathological, psychological, and also biological processes that exhibit dynamic processes. Mental disorders arise when various symptoms actively maintain and reinforce each other. External factors (e.g., critical life events or experiential manipulations) can influence the network, activating symptoms within the network that subsequently lead to changes in downstream, adjacent symptoms (Borsboom, 2017). Considering symptoms and therefore data from different domains may be advantageous due to the biopsychosocial nature of mental disorders (for recent examples of multimodal integration, see: e.g., Astill Wright et al., 2021; Stein et al., 2023; Tao et al., 2022). Given the multimodal nature of mental disorders, a unified theoretical framework seems to be required to understand mental disorders comprehensively. The systems approach is not constrained by specific data types or mechanisms, making it a useful meta-theory for integrating diverse observations and simultaneously studying multiple modalities (Borsboom, 2017; Medaglia et al., 2017). It fosters interdisciplinary collaboration by allowing the simultaneous examination of various modalities through approaches like multilayer networks, integrated networks, or network-based regressor networks (Blanken et al., 2021; Brooks et al., 2020). Emphasizing the interconnected nature of individual symptoms, mental processes, and (neurobiological) mechanisms, the systems perspective serves as a meta-theory for future scientific endeavors (Blanken et al., 2021; Taschereau-Dumouchel et al., 2022).

The systems approach not only integrates symptom areas from supposedly different diagnoses or modalities into a single disorder model. It can also be applied to different models of diagnostic and intervention approaches, such as the Extended Evolutionary Meta Model (EEMM; Hayes et al., 2020). The EEMM applies evolutionary concepts of context-appropriate variation, selection, and retention to relevant biopsychosocial processes, such as cognition, affect, or physiology. It also emphasizes connections and interactions among psychological constructs and describes mental disorders as the result of a process of maladaptation from an evolutionary standpoint. As a framework for diagnostic and intervention approaches, the EEMM can accommodate any set of psychological or psychopathological processes, regardless of specific therapy orientations. The EEMM as a theoretical model encourages a new form of process-based functional analysis and therapy, such as Process-based Therapy (PBT; Hofmann & Hayes, 2019), which can be put into practice with the help of concepts and analytic methods of the systems approach.

The transdiagnostic nature of the systems approach enables the integration of different therapeutic orientations and their principles and could thus enrich therapeutic practice. Network analysis, for example, aligns with more traditional behavior therapy or newer process-based approaches, such as PBT (Hofmann & Hayes, 2019), by integrating functional analysis with data-driven techniques, such as EMA. Considering complex interrelationships between psychological and psychopathological processes, potentially relevant characteristics of the individual or disorder are identified (Hayes et al., 2020). Network analysis can assist in examining the interrelatedness of these processes to recognize causal relationships, quantify preliminary analysis, and finalize the conceptual functional analysis (see S. C. Hayes, Hofmann, Hayes et al., 2020b for additional information about functional analysis). The systems approach facilitates the integration of clinical knowledge and data-based approaches, which could ultimately lead to case formulations with personalized network assessment (e.g., Burger et al., 2022) and targeting empirically established processes that are functionally important to the individual (Ong et al., 2022). Importantly, the systems approach does not contradict existing concepts of mental disorders but rather provides impetus for the integration and expansion of existing approaches.

It is crucial to emphasize that the systems perspective as a meta-theory also does not inherently represent an alternative to the existing diagnostic and classification approach or to established group-based research in the field. It is important to consider that current classificatory systems serve multiple functions. Beyond scientific validity, for example, they also address international comparison of mental disorders, taking into account public health and policy perspectives (for a brief discussion, see: Rief et al., 2023). Instead, the systems approach prompts an adjusted perspective on the nature and therapy of mental disorders that could enrich and expand our current understanding while coexisting within established principles and classificatory systems. Should this new perspective expand our understanding of mental health and introduce novel insights, it may encourage refinements in approaches to diagnosis and classification in the future.

Graph Theory and Control Theory are toolboxes used within the systems approach that facilitate the modeling and analysis of network structures (Bollobás, 2012). Graph Theory provides insights into network topology, properties, and multivariate dependencies (Barnes & Harary, 1983; Newman, 2010), while Control Theory enables the quantitative assessment of a node’s control over the system, predicting temporal patterns, and guiding interventions (Stocker et al., 2023). Graph Theory, a branch of discrete mathematics, analyzes complex networks represented as graphs with nodes and edges. Nodes represent variables, while edges depict relationships. Directed edges indicate one-way connections and undirected edges represent mutual relationships. Centrality metrics (e.g., Epskamp & Fried, 2018; Hofmann et al., 2016), such as degree centrality and expected influence, identify influential nodes and provide information about network structure and dynamics (for a detailed description, see: Robinaugh et al., 2016). Given the multimodal nature of the data, understanding the composition and influences of control points within the network is crucial. Within the systems approach, several statistical methods have been utilized to effectively ascertain network structures between diverse nodes of mental disorders (Bringmann et al., 2013; Epskamp et al., 2012; Haslbeck & Waldorp, 2020).

Control Theory (Hyland, 1987), originally developed in engineering (e.g., Xiao & Gao, 2010), models complex system dynamics, analyzing functional relationships, resilience, and control (Liu et al., 2011; Liu & Barabási, 2016). Network control theory focuses on the effects of inputs on the network system, dynamics over time, and identifies nodes exerting significant control over the organization (Hahn et al., 2021; Stocker et al., 2023). Different metrics, such as average controllability (e.g., Gu et al., 2015; Henry et al., 2022), modal controllability (Scheffer, 2010), and boundary controllability (Hahn et al., 2021), describe the ability of a network node to control the rest of the system, enabling targeted interventions. Our approach will utilize topological measures and control theory in conjunction with longitudinal and multimodal data (e.g., Goldstein-Piekarski et al., 2022). This allows for a comprehensive understanding of network dynamics and enables interventions such as psychological, pharmacological, or neurostimulatory approaches to induce changes in the initial state of the system (Henry et al., 2022; Medaglia et al., 2017).

In summary, the systems approach views mental disorders as interconnected networks of symptoms, providing a meta-theoretical framework for integration across modalities. The usage of control theory and graph theory as descriptive methodologies is critical to understanding the complex networks that underlie mental disorders. Graph theory facilitates insights into network structure and dynamics, while control theory enables the quantification of specific node influences.

### Addressing Problem Two. A Deflationary Stance Towards Comorbidity

As previously mentioned, comorbidity can be seen as a consequence of artificially segmenting a complex clinical landscape into multiple components (Maj, 2005). MMDNs describe individual symptoms as a part of different, complex, time-varying disorder patterns without a one-to-one correspondence to diagnostic boundaries. By conceptualizing disorders as clusters of directly related symptoms, artificial boundaries between disorders do not need to be drawn and comorbidity itself vanishes (e.g., Köhne & Isvoranu, 2021). Recognizing mental disorders as a complex landscape without clear syndromes or categorically defined symptom patterns allows for a broader understanding of symptom relationships without making a priori assumptions (Borsboom, 2008; Borsboom & Cramer, 2013).

Mental disorders can be understood as networks of interacting symptoms, where a single symptom can influence and is influenced by symptoms traditionally assigned to different diagnostic categories (Levinson et al., 2017). A patient may be self-critical, leading to dissatisfaction with one’s body image. This leads to deep sadness and crying, which, e.g., reinforces food restrictions. Dieting in turn leads to anhedonia and fatigue. Thus, a network analysis of this patient would represent symptoms that are typically assigned to different categories (namely depression and eating disorders). Networks, being transdiagnostic in nature, can map out symptom combinations and pathological processes (like rumination or anhedonia: McLaughlin & Nolen-Hoeksema, 2011; Mendoza et al., 2022; Rutherford et al., 2023). Furthermore, the utilization of longitudinal network analyses allows for the identification of transdiagnostic features, such as bridge symptoms, that may have relevance across different configurations of mental disorders (Fried et al., 2017; Jones et al., 2021). For instance, *self-blame* is situated within a problem domain resembling depression, characterized by feelings of worthlessness, as well as within a problem domain associated with cognitive performance deficits. Therefore, *self-blame* plays a significant role in individuals experiencing depressive symptoms as well as cognitive performance impairments (Blanken et al., 2018). Ultimately, the approach allows for the investigation of underlying mechanisms contributing to the frequent co-occurrence of specific disorders, while also capturing temporal fluctuations in symptom associations, thereby providing valuable insights into the etiology and dynamics of putative “comorbidity” (Jones et al., 2021).

The identification of bridge symptoms in various configurations of mental disorders could facilitate research into transdiagnostic processes. It allows us to focus on identifying underlying processes that cause and maintain multiple “comorbid” disorders (e.g., Barlow et al., 2004; Dalgleish et al., 2020; Insel et al., 2010; Seligman, 2014). In turn, we can use this knowledge to adapt therapeutic strategies originally developed for distinct disorders and view them as a toolbox that can be utilized whenever the network dynamic of a given patient suggests clinical utility (e.g., Boswell et al., 2014; Norton & Barrera, 2012).

To summarize, combining the systems perspective with multimodal and longitudinal measures dismantles the “problem” of comorbidity as a pseudo-problem that should be viewed from a deflationary standpoint. By seeing mental disorders as sets of interacting symptoms, the “problem” of comorbidity vanishes. To summarize: We can achieve a more comprehensive and nuanced understanding of mental disorders by considering interconnected symptoms that go beyond conventional diagnostic categories. This opens up new avenues for the development of interventions targeting common underlying mechanisms among multiple disorders rather than relying solely on diagnostic labels.

### Addressing Problems Three and Four. Dealing with Non-Ergodicity and Capturing Heterogeneity

As mentioned, traditional psychometric procedures are limited in accurately representing mental disorders and therapeutic processes due to unrealistic ergodic assumptions. Within traditional perspectives, individual variability is often considered as “error variance”, leading to a loss of information regarding individual patterns and their contextual or treatment-related associations (Molenaar, 2008). Due to the presence of heterogeneity both between individuals and within individuals (Molenaar, 2008, 2013), and the limitations in identifying distinct symptom patterns within diagnostic labels (e.g., Contractor et al., 2017), we propose the utilization of dynamical network analyses which consider individual variability and aim to minimize the impact of aggregated group patterns (Fisher et al., 2018; Molenaar, 2013; Wright & Woods, 2020). Dynamic networks visualize the direction of relationships between nodes and provide insights into Granger causality (Granger, 1969), which assumes that knowledge of a system’s status at earlier time points aids in predicting its future behavior (Epskamp et al., 2018b; Granger, 1969). Granger causality can be instrumental in prioritizing specific symptoms within interventions by identifying their role in driving network dynamics (e.g., Goldin et al., 2014; Hamilton et al., 2011; Zuidersma et al., 2022). Furthermore, it may enhance treatment planning by enabling prediction of future behavior of a given network and by identifying time points for effective interventions (e.g., Bose et al., 2017; Goldin et al., 2014; Mai et al., 2021).

In the context of network analysis, heterogeneity is analyzed by estimating *within-person individual networks* (e.g., Borsboom et al., 2021). Accordingly, each participant is intensively studied at numerous time points, followed by person-specific analyses. Extensive longitudinal datasets are collected to fit network models, such as vector autoregressive models (VAR) that emphasize within-person effects and account for individual variations in the manifestation of disorder over time (Beck & Jackson, 2020; Fried & Cramer, 2017). EMA (Shiffman et al., 2008) is a valuable tool for collecting relevant variables (e.g., symptoms) multiple times throughout daily life, capturing their dynamic relationships. For example, EMA can be used to assess contentment and cheerfulness dynamics during the day. By employing individual VAR models, we can analyze the contemporaneous co-occurrence of symptoms and explore temporal patterns, such as how satisfaction predicts serenity at subsequent time points (Curtiss et al., 2022). Multilevel VAR models extend these analyses to a group of individuals, differentiating within-individual dynamics from stable inter-individual differences. These models provide individual-specific directed networks, a group-level network, and a variability network indicating the degree of variation in networks across individuals within the group (Bringmann et al., 2013).

By examining relationships between symptoms at the individual level, we can make statements about symptom patterns at the group level while considering individual-specific manifestations (Gates & Molenaar, 2012). One method that allows for this analysis while circumventing the ergodic error is GIMME, which uses a unified structural equation model (uSEM; Kim et al., 2007). GIMME identifies non-generalizable relationships between symptoms for each individual, considered heterogeneous, and in an iterative process integrates pathways applicable at the group levels if they improve individual fit. Using such integrated pathways, the algorithm can discover generalizable components (i.e., typical patterns of e.g., symptoms) within a given population (Hoekstra et al., 2022; Lane & Gates, 2017).

A simpler alternative to the bottom-up GIMME approach might be to utilize panel VAR models if a group of subjects needs to be studied over time. Panel VAR can be used to model dynamic associations among several symptoms within a cohort over time. For example, it could be of interest to investigate the extent to which associations between symptoms within a cohort change at the beginning of therapy, post-therapy, and at a follow-up appointment. Panel VAR models are therefore an extension of VAR models that include a cross-sectional dimension. They differentiate between within-subject and between-subject effects by combining cross-sectional data and time series measurements of the same individuals at different time points (Hamaker et al., 2015). Panel networks can be estimated with limited measurements using cross-lagged models with random intercepts (e.g., Epskamp, 2020). They serve as within-person versions of stationary cross-lagged panel models based on densely measured longitudinal data. Lastly, it is possible to generate group-level hypotheses based on heterogeneous intraindividual data, even with cross-sectional networks. Network mixture models or machine learning algorithms (Bzdok & Meyer-Lindenberg, 2018) can identify subgroups of individuals exhibiting higher homogeneity in their respective group-level networks (Ten Have et al., 2016). For example, in a cross-sectional survey of a patient cohort, subgroups could be identified that show comparable associations between symptoms (for a brief discussion, see: Fried & Cramer, 2017). Thus, the systems approach provides methods tailored to various research questions, which consider heterogeneity within and between individuals.

### Addressing Problem Five. Advancing our Understanding of Causal Processes

To gain a deeper understanding of psychopathology, it seems crucial to elucidate the causal relationships among symptoms (Schmittmann et al., 2013). Cross-sectional data and undirected correlation networks have considerable limitations for establishing causal inferences (e.g., Contreras et al., 2019; Malgaroli et al., 2021; Robinaugh et al., 2020). The inference of causal relationships between symptoms can be seen as the ability to modify the probability distribution of one symptom by altering the state of another symptom through experimental or natural interventions (Borsboom, 2017). We advocate for dynamic analyses due to the limitations of cross-sectional analyses in estimating the directionality of edges, which indicate temporal precedence, a necessary condition for causality (e.g., Thoemmes, 2015).

Understanding network dynamics involves considering carry-over effects resulting from repeated measurements. Temporal order is essential for causal interpretation, as it demonstrates the temporal priority of an effect (Bringmann et al., 2013). Several established methods, such as Granger causality (Granger, 1969) or Bayesian networks (Pearl & Mackenzie, 2019), can be used to characterize necessary (but not always sufficient) features of causal systems.

Generally, Bayesian networks are probabilistic graphical models that represent variables and their conditional interdependencies through a directed and acyclic graph. They excel in predicting the likelihood of a known cause being causally responsible for an observed event (e.g., Herzog et al., 2022). Unveiling causal relationships between variables helps establish the directionality of relationships and enables the identification and distinction between epiphenomena and primary causal factors. Causal relationships imply that changes in one variable cause changes in another, while epiphenomena are secondary phenomena not causally linked to the primary causal factor but may appear correlated. Understanding causality allows for the differentiation between variables that have a causal impact in a given network and those that are coincidental or influenced by other underlying factors. In categorical models, epiphenomena are included and treated similarly to potentially causal features, as current diagnostic systems do not prioritize some symptoms over others. Epiphenomena may even have the greatest prominence in the observed pattern of disorder in a patient and become a primary focus during therapy. However, using MMDNs, we aim to differentiate between epiphenomena and causal processes, thus identifying promising intervention targets and temporal windows of opportunity.

As for overcoming problems with ergodicity, the GIMME algorithm (Gates & Molenaar, 2012) can also be applied to identify causal factors. By considering the temporal dependencies between variables, GIMME provides insights into temporal precedence and the directionality of influence between variables. The temporal dimension enhances our understanding of how causal factors unfold and impact the network structure, contributing to a deeper comprehension of complex systems and their underlying mechanisms. Furthermore, GIMME provides information about causal factors by drawing conclusions about the group (nomothetic) based on individual-specific data (idiographic). GIMME has the assumption that people share similar representations of causal structures, improving the accuracy and effectiveness of its inference procedure. Thus, GIMME integrates individual-level causal structures with group-level structures, generating individual causal models that incorporate shared structures within the group. This statistical approach enables the bottom-up identification of group-level causal structures using idiographic data.

The systems approach as a framework can be used in this context to generate and test hypotheses. Associations among relevant problems can be analyzed within a specific sample, starting with a particular disorder (e.g., social anxiety disorder), undergoing specialized treatment (comparison of therapy vs. control group), or exhibiting specific characteristics (e.g., non-responders). The selection of nodes representing problems in the network should be theory-driven, for example, incorporating cognitive, affective, and attention-related processes in a sample of patients with social anxiety disorder, aligning with existing disorder models. Within the framework of the systems approach, GIMME can be employed on the one hand to draw generalized conclusions about the associations of processes within the patient sample. However, the analysis does not have to be limited to a supposedly definable disorder. By incorporating patients with supposedly multiple diagnoses, we can identify underlying processes contributing to comorbidity beyond symptom-symptom connections (i.e., bridging connections). Therefore, comorbidity arises not only from shared symptoms but also from symptoms indicative of the same underlying process or mechanism (Cramer et al., 2010). Identifying underlying processes at the group level could facilitate the identification of causal processes relevant to the maintenance of multiple disorders. Beyond drawing generalized conclusions at the group level, GIMME takes into account associations at the level of the individual. This approach enables the identification of connections that are unique to one or a subgroup of individuals, which can be particularly relevant given heterogeneous samples (Lane et al., 2019). Once data-driven associations between problems have become clear through obtained networks, these findings, in turn, stimulate the generation of new hypotheses. These hypotheses can be tested in subsequent studies, such as through targeted perturbation of the network (e.g., addressing the problem identified as central in the sample of socially anxious patients).

As mentioned, modern algorithms model candidates for causal links by identifying central symptoms, which serve as indicators of influential nodes, as changing them may have beneficial downstream effects (Hofmann et al., 2016; Levinson et al., 2022; Rhemtulla et al., 2016; Rodebaugh et al., 2018). Symptoms with high centrality could demonstrate a strong capacity to forecast long-term trajectories under some circumstances (Elliott et al., 2020). However, in undirected cross-sectional network studies on depression, there is substantial variability in the data as well as contradictory findings regarding centrality measures, with no single symptom consistently identified as the most central (Huang et al., 2023). Therefore, the use of MMDNs to capture *crucial nodes* and their *temporal changes* may become useful to address the limitations of cross-sectional network studies.

### Limitations and Future Directions

We anticipate that incorporating the temporal dimension within the proposed approach will significantly enhance our understanding of symptom networks. However, the relationship between causal influence and nodal centrality measures is not straightforward and is sometimes weak, except for eigenvector centrality (Borsboom et al., 2021; Dablander & Hinne, 2019; Spiller et al., 2020). Therefore, it is crucial to interpret centrality measures cautiously and refrain from attributing causal implications to them. The impact of seemingly central nodes should be understood as relative importance within the network, distinct from causal influence, which requires empirical substantiation. Future studies should be designed to enhance the ability to establish causal relationships between symptoms. This requires the use of longitudinal data and novel analytic techniques, as well as the evaluation of assumptions for causal inference, such as accounting for sources of non-replacement or violation of positivity (Huang et al., 2023).

Furthermore, it is crucial to bear in mind that the majority of previous analysis models operate under the assumption of a constant network structure, without accounting for potential changes of mean or variance over time (i.e., stationarity). However, assuming that all individuals are influenced by the same processes over time is implausible, given the dynamic nature of psychopathology, interventions, and psychotherapeutic processes. To drop the assumption of stationarity, we can utilize novel time-varying network models in future studies, which capture *variations in associations* between variables over time (Siepe et al., 2024). It is important to elaborate on this point. Theoretical work such as Hofmann et al. (2016) often implicitly or explicitly assumes a changing network topology and connection strength, while the majority of contemporary empirical network studies estimate networks that have a constant structure. This point is true for both nomothetic and ideographic network approaches. For both types of network analysis, we would like to use methods that can map dynamic structural changes. Therefore, to enable the full potential of network approaches in empirical science, methodological developments are necessary.

Additionally, temporal networks often suffer from data scarcity (e.g., Epskamp, Borsboom et al., 2018). The available data points are insufficient to capture the complexity and dynamics of the network fully. This limitation can result in limited temporal resolution, making it challenging to accurately estimate network properties (for a brief discussion, see: Jordan et al., 2020). Understanding measurement error allows researchers to construct sophisticated temporal network models, but challenges remain, including limited availability and quantity of EMA data and the need for advancements in mathematical modeling, necessitating future progress in both areas. Passive data measurement methods, like speech transcription or screen time of smartphones (for a deeper discussion, see: Myin-Germeys & Kuppens, 2021), hold promise for generating larger datasets without burdening participants excessively (e.g., Christensen et al., 2009).

Diagnostic systems have goals beyond mapping and describing variables. They must also be structured to remain understandable for clinicians, politicians, and the general population (e.g., Rief et al., 2023). If MMDNs can indeed provide a better explanation of psychopathology and facilitate improved treatment options, it is important to develop a clear and understandable terminology that can be easily understood by a wide range of people. At the moment, however, it seems crucial to state that our ideas are intended for research contexts first and foremost. Time and data will tell whether new conceptualizations of psychopathology will enhance, partially replace, or have a limited long-term impact on the diagnosis and treatment of mental disorders.

We acknowledge that enhanced diagnostics may not automatically translate into improved treatment outcomes. Nevertheless, refining diagnostic procedures is a lever that fosters optimism for advancements in clinical psychology. However, the potential clinical advantages of network models in therapy require more empirical support in the coming years. Suggestions for treatment planning (e.g., Levinson et al., 2021) and initial single-case studies, such as the use of process-based therapy (Ong et al., 2022) or approaches for combining case conceptualization with personalized network estimation (Burger et al., 2022) appear promising. Yet, larger therapy studies are needed in the future to verify the potential clinical benefits for practitioners as well as to demonstrate potential improvements in the effectiveness of treating various mental disorders.

## Conclusion

MMDNs can offer a promising opportunity for understanding mental disorders and developing more efficacious treatments. This emphasis is evident within the framework of the upcoming project *Dynamic Network Approach of Mental Health to Stimulate Innovations for Change*. We expound upon methodologies, which form a constituent but not an exhaustive aspect of the DYNAMIC initiative. As stated, these research approaches combine longitudinal measurements, multimodal sources of data, and causal inference techniques. Longitudinal studies and data collection methods enable the modeling of network states over time, leading to an enhanced understanding of mental health and treatment outcomes. The systems approach provides a meta-theory that integrates diverse models and explanatory approaches, allowing for the study of interconnected symptoms, processes, and mechanisms within a network. By adopting the systems perspective, comorbidity can be understood as clusters of symptoms, facilitating research into transdiagnostic processes. We aim to address the issue of heterogeneity in mental disorders by capturing individual variability and allowing for personalized analysis. Individual analysis of longitudinal data tackles the ergodicity problem and provides insights into the temporal (and hopefully causal) dynamics of symptoms. Overall, MMDNs can contribute to a more nuanced understanding of mental health and offer the potential for improved diagnosis, treatment, and intervention strategies to suffering and helping address one of the most pressing societal issues to date.
